# Supplementary material for: Analysis of PPARγ Signaling Activity in Psoriasis
Source: Int J Mol Sci. 2021 Aug 10;22(16):8603. doi: 10.3390/ijms22168603 (PMC8395241; doi:10.3390/ijms22168603)
Supplement: Supplementary file 1 [file ijms-22-08603-s001.zip › Supplemental materials_Analysis of PPARg signaling activity in psoriasis/Pathway models/Models images and html files/Differentiation of psoriatic T cells/6192.html]

IFNG


# Protein IFNG

|  |  |
| --- | --- |
| URN | urn:agi-llid:3458 |
| Total Entities | 0 |
| Connectivity | 16299 |
| Name | IFNG |
| Description | interferon, gamma |
| Notes | This gene encodes a soluble cytokine that is a member of the type II interferon class. The encoded protein is secreted by cells of both the innate and adaptive immune systems. The active protein is a homodimer that binds to the interferon gamma receptor which triggers a cellular response to viral and microbial infections. Mutations in this gene are associated with an increased susceptibility to viral, bacterial and parasitic infections and to several autoimmune diseases. [provided by RefSeq, Dec 2015] |
| Primary Cell Localization | Extracellular |
| Class | Ligand |
| ObjectType | Protein |

---

|  |  |
| --- | --- |
| Pathway | MHC2-Mediated Antigen Presentation |
|  | MHC1-Mediated Antigen Presentation |
|  | Natural Killer Cell Activation through C-Type Lectin-like Receptors |
|  | Natural Killer Cell Activation through ITAM-Containing Receptors |
|  | Natural Killer Cell Activation through ITSM-Containing Receptors |
|  | Natural Killer Cell Activation |
|  | CD8+ T-Cell Activation |
|  | T-Cell Cytotoxic Mediated Cell Death |
|  | Th1-Cell Differentiation |
|  | T-Cell Receptor Signaling |
|  | AHR Signaling in Treg and Dendritic Cells Function |
|  | AHR Signaling in Tr1 Cells Function |
|  | Mast-Cells De Novo Synthesized Mediators via IgE Independent Signaling |
|  | Macrophage M1 Lineage |
|  | AHR Signaling in M1 Macrophages Function |
|  | Histidine-Rich Glycoprotein (HRG) |
|  | Proteins Involved in Atherosclerosis |
|  | Dendritic Cells Function in Atherosclerosis |
|  | Th1-Cells Activation and Proliferation in Atherosclerosis |
|  | Proteins Involved in Dilated Cardiomyopathy |
|  | Lymphocyte Mediated Myocardial Injury in Myocarditis |
|  | Proteins Involved in Myocarditis |
|  | SOCS in Negative Feedback Regulation in Myocarditis |
|  | Proteins Involved in Acne Vulgaris |
|  | Atopic Dermatitis Onset |
|  | Proteins with Altered Expression in Atopic Dermatitis |
|  | Proteins Involved in Atopic Dermatitis |
|  | Atopic Dermatitis |
|  | Proteins Involved in Psoriasis |
|  | T-Cells Differentiation Block in Psoriasis |
|  | IFN-gamma/TNF-alpha Mediated Cell Proliferation in Psoriasis |
|  | Th0-Cell Aberrant Activation in Systemic Lupus Erythematosus |
|  | Proteins Involved in Systemic Lupus Erythematosus |
|  | Th1-Cell Function in Systemic Lupus Erythematosus |
|  | Proteins Involved in Vitiligo |
|  | Acute Cytotoxic CD8+T-Cell Response against Melanocytes |
|  | Vitiligo |
|  | T-Cell Cytotoxic Response against Melanocytes in Vitiligo |
|  | CD8+ T-Cell Response to Self-Determinants in Diabetes |
|  | Natural Killer T-Cell Roles in Diabetes Mellitus Type 1 |
|  | non-Suppressive Treg-Cell in Diabetes Mellitus Type 1 |
|  | Natural Killer Cell in Diabetes Mellitus Type 1 |
|  | Toll-like Receptors in beta-Cell |
|  | Proteins Involved in Diabetes Mellitus Type 1 |
|  | Proteins Involved in Diabetes Mellitus Type 2 |
|  | Proteins Involved in Insulin Resistance |
|  | beta-Cell Destruction through Cytokines in Diabetes Mellitus |
|  | beta-Cell Destruction in Diabetes Mellitus Type 1 |
|  | Nitric Oxide Effects on beta-Cell |
|  | Immune Sustem Activation in Hashimoto's Thyroiditis |
|  | Proteins Involved in Hashimoto's Thyroiditis |
|  | Hashimoto's Thyroiditis |
|  | Apoptosis of Thyroid Cell in Hashimoto's Thyroiditis |
|  | Proteins Involved in Graves Disease |
|  | Graves Ophthalmopathy |
|  | Proteins Involved in Hypothyroidism |
|  | Proteins Involved in Myasthenia Gravis |
|  | Proteins Involved in Celiac Disease |
|  | CD4+T-Cell Response in Celiac Disease |
|  | CD8+ T-Cell Response in Celiac Disease |
|  | Proteins with Altered Expression in Celiac Disease |
|  | Celiac Disease |
|  | Metastatic Colorectal Cancer |
|  | Crohn's Disease |
|  | Th1-Cell Activation in Crohn's Disease |
|  | Macrophage Dysfunction |
|  | Proteins Involved in Inflammatory Bowel Diseases |
|  | Proteins Involved in Helicobacter Infections |
|  | Proteins Involved in Ulcerative Colitis |
|  | Th2-Cell Function in Ulcerative Colitis |
|  | Proteins Involved in B-Cell Chronic Lymphocytic Leukemia |
|  | Proteins Involved in Multiple Myeloma |
|  | Proteins Involved in Hepatocellular Carcinoma |
|  | Proteins Involved in Pancreatic Cancer |
|  | ADAM33 Role in Asthma |
|  | Proteins Involved in Cystic Fibrosis |
|  | Th17-Cell and Th1 Immune Responsein Psoriatic Arthritis |
|  | Keratinocyte Activation in Psoriatic Arthritis |
|  | Proteins Involved in Psoriatic Arthritis |
|  | PTPN22 Role in Rheumatoid Arthritis |
|  | Proteins Involved in Rheumatoid Arthritis |
|  | FAS Mediated Apoptosis in Systemic Scleroderma |
|  | Proteins Involved in Systemic Scleroderma |
|  | Proteins Involved in HIV Type 1 Infection |
|  | CD8+ T-Cell Action Impairment in HIV Type 1 Infection |
|  | Proteins Involved in Periodontitis |
|  | beta-Casomorphins/Opioid Receptors Affect Brain Function (Hypothesis) |
|  | Bone Resorption by Osteoclasts in Osteoporosis |
|  | Proteins with Altered Expression in Psoriatic Arthritis |
|  | Proteins with Altered Expression in Rheumatoid Arthritis |
|  | Proteins Involved in Glioblastoma |
|  | Proteins Involved in Astrocytoma |
|  | Proteins Involved in Glioma |
|  | Proteins Involved in Medulloblastoma |
|  | Proteins Involved in Neuroblastoma |
|  | MBP/MOG/PLP in Immune System Activation |
|  | Proteins Involved in Multiple Sclerosis |
|  | IL6/IL12 Signaling Activates Immune System in Multiple Sclerosis |
|  | Proteins Involved in Cataract |
|  | Proteins Involved in Chronic Obstructive Pulmonary Disease |
|  | T-Cell Cytotoxic Activation |
|  | Proteins Involved in Pulmonary Emphysema |
|  | Proteins Involved in Glomerulonephritis |
|  | Proteins Involved in Male Infertility |
|  | HMGB1 Expression Targets |
|  | BMP4/BMPR2 Expression Targets |
|  | IFNG -> ARRB1/STAT1 Signaling |
|  | CD2 Expression Targets |
|  | CD81 -> AP-1 Expression Targets |
|  | FCGR3A Expression Targets |
|  | CD72 -> CREB/CREBBP Expression Targets |
|  | CD8 -> ATF/CREB/CREBBP Expression Targets |
|  | CD8 -> NF-kB Expression Targets |
|  | CD8 -> STAT Expression Targets |
|  | CD80 -> AP-1 Expression Targets |
|  | CD81 Expression Targets |
|  | CD80 -> NF-kB Expression Targets |
|  | CXCL2 Expression Targets |
|  | CSF2 -> NF-kB Expression Targets |
|  | CSF2 -> STAT Expression Targets |
|  | Clozapine Induced Granulocytopenia |
|  | NCAM1 Expression Targets |
|  | FGF2 -> AP-1/CREB/CREBBP/ELK/SRF/MYC Expression Targets |
|  | Elevated Receptors -> Expression Targets in Colon |
|  | Elevated Receptors -> Expression Targets in Appendix |
|  | GH1/GHR -> NF-kB/ELK/SRF/MYC Expression Targets |
|  | GH1/GHR -> STAT Expression Targets |
|  | GH1/PRLR Expression Targets |
|  | IL12 Antitumor Effect is Blocked in Cancer |
|  | MHC1 Causes Antigen Presentation Failure in Cancer Immune Escape |
|  | Myeloid Derived Suppressor Cells in Cancer Immune Escape |
|  | IDO1 in Cancer Immune Escape |
|  | Effector T-cell Inactivation in Cancer Immune Escape |
|  | IGF1 -> MEF/MYOD/MYOG Expression Targets |
|  | IGF1 -> STAT Expression Targets |
|  | IGF1 -> ELK/SRF/HIF1A/MYC/SREBF Expression Targets |
|  | INHBA/ACVR2/BMPR Expression Targets |
|  | IFNA1/IFNR Expression Targets |
|  | IFNG/IFNR Expression Targets |
|  | IL7 Expression Targets |
|  | IL21 Expression Targets |
|  | IL15 Expression Targets |
|  | IL12B Expression Targets |
|  | IL3 Expression Targets |
|  | IL2 Expression Targets |
|  | Leptin -> STAT Expression Targets |
|  | Leptin -> ELK/SRF Expression Targets |
|  | Prostaglandin F Expression Targets |
|  | LDLR -> Expression Targets in Lymphoid System and Blood |
|  | Elevated Receptors -> Expression Targets in Lymphoid System and Blood |
|  | CD Markers -> Expression Targets in Lymphoid System and Blood |
|  | VIPR1/VIPR2 -> Expression Targets in Lymphoid System and Blood |
|  | GPCRs Family -> Expression Targets in Lymphoid System and Blood |
|  | Cell Adhesion Receptors -> Expression Targets in Lymphoid System and Blood |
|  | Immunoglobulin Superfamily -> Expression Targets in Lymphoid System and Blood |
|  | Receptor Tyrosine Kinase Family -> Expression Targets in Lymphoid System and Blood |
|  | Elevated Receptors -> Expression Targets in Bone Marrow |
|  | Interleukin Receptors -> Expression Targets in Lymphoid System and Blood |
|  | CD40 -> Expression Targets in Thymus |
|  | CD40 -> Expression Targets in Lymph Node |
|  | C5AR1 -> Expression Targets in Lymphoid System and Blood |
|  | IgE Receptors -> Targets in Lymphoid System and Blood |
|  | NGF -> SMAD3/NF-kB Expression Targets |
|  | DLL1 Expression Targets |
|  | NOTCH Expression Targets |
|  | Adenosine Expression Targets |
|  | GNRH1 Expression Targets |
|  | NTS Expression Targets |
|  | AGT -> CREB Expression Targets |
|  | AVP/Gq -> STAT Expression Targets |
|  | IFNA1/Gq Expression Targets |
|  | TAC1 Expression Targets |
|  | AVP/Gq -> MEF/MYOD/NFATC/MYOG Expression Targets |
|  | AGT -> STAT Expression Targets |
|  | PRL/PRLR Expression Targets |
|  | PRL/GHR -> STAT Expression Targets |
|  | PRL/GHR -> NF/kB/ELK/SRF/MYC Expression Targets |
|  | PDCD1 -> ATF/CREB/CREBBP Expression Targets |
|  | PDCD1 -> STAT Expression Targets |
|  | PDCD1 -> NF-kB Expression Targets |
|  | PDCD1 -> NFATC Expression Targets |
|  | PDCD1 -> AP-1 Expression Targets |
|  | Collagen -> NF-kB Expression Targets |
|  | FLT3LG -> AP-1/CREB/CREBBP Expression Targets |
|  | Elevated Receptors -> Expression Targets in Uterus |
|  | Elevated Receptors -> Expression Targets in Lung |
|  | Ras-GAP Regulation Signaling |
|  | TCR -> CREB/CREBBP/ATF Expression Targets |
|  | TCR -> NF-kB Expression Targets |
|  | TCR -> NFAT Expression Targets |
|  | TCR -> AP-1 Expression Targets |
|  | TGFB2-TGFBR1 Expression Targets |
|  | TGFB3-TGFBR1 Expression Targets |
|  | TGFB1-TGFBR1 Expression Targets |
|  | TGFB1-ACVRL1 Expression Targets |
|  | TNFSF14 Expression Targets |
|  | TNF -> AP-1 Expression Targets |
|  | CD72 -> AP-1 Expression Targets |
|  | CD40LG -> NF-kB/ELK/SRF -> CREB/NFATC Expression Targets |
|  | TLR3 Expression Targets |
|  | TLR4 -> AP-1 Expression Targets |
|  | TLR5 Expression Targets |
|  | TLR7 Expression Targets |
|  | TLR1 -> 2/6 Expression Targets |
|  | TLR4 -> NF-kB/IRF Expression Targets |
|  | IL1A Expression Targets |
|  | TLR9 Expression Targets |
|  | ROS in Triggering Vascular Inflammation |
|  | WNT5A Expression Targets |
|  | WNT7A Expression Targets |
|  | cypa |
|  | PPAR Psoriasis |
|  | prarg negative regulators, ps-positive |
|  | pprarg neg, uknown targets, ps-positive |
|  | prarg neg,ukn expres targets, ps-positive |
|  | PPARG negative regulators |
|  | PPARG negative targets |
|  | pparg expr target\_regulators |
|  | Neighbors of disease exacerbation |
|  | Neighbors of tumor progression |
|  | Neighbors of cancer progression |
|  | Neighbors of inflammatory status |
|  | Neighbors of overall survival |
|  | Neighbors of disease severity |
|  | Neighbors of tumor size |
|  | Neighbors of lesion size |
|  | Neighbors of mortality |
|  | Neighbors of survival rate |
|  | Neighbors of infarct size |
|  | Neighbors of survival time |
|  | Neighbors of functional recovery |
|  | Neighbors of insulin sensitivity |
|  | Neighbors of bacterial load |
|  | Neighbors of cancer survival |
|  | Neighbors of graft survival |
|  | Neighbors of therapeutic efficacy |
|  | Neighbors of clinical stage |
|  | Neighbors of lethality |
|  | Neighbors of tumor regression |
|  | Neighbors of recurrence-free survival |
|  | Neighbors of disease-free survival |
|  | Neighbors of tumor response |
|  | Neighbors of macrophage count |
|  | Neighbors of long-term survival |
|  | Neighbors of treatment outcome |
|  | Neighbors of bone mass |
|  | Neighbors of body weight |
|  | Neighbors of body weight gain |
|  | Neighbors of cardiovascular risk |
|  | Neighbors of microvessel density |
|  | Neighbors of tumor recurrence |
|  | Neighbors of tumor promotion |
|  | Neighbors of neutrophil count |
|  | Neighbors of glucose tolerance |
|  | Neighbors of morbidity |
|  | Neighbors of fibrosis progression |
|  | Neighbors of mortality rate |
|  | Neighbors of fat mass |
|  | Neighbors of exhaustion |
|  | Neighbors of bone mineral density |
|  | Neighbors of adverse outcome |
|  | Neighbors of lesion area |
|  | Neighbors of cell density |
|  | Neighbors of progression-free survival |
|  | Neighbors of apoptosis rate |
|  | Neighbors of early mortality |
|  | Neighbors of viral load |
|  | Neighbors of blood pressure |
|  | Neighbors of leukocyte count |
|  | Neighbors of vascular reactivity |
|  | Neighbors of vascular leakage |
|  | Neighbors of body mass index |
|  | Neighbors of vascular density |
|  | Neighbors of muscle mass |
|  | Neighbors of cancer incidence |
|  | Neighbors of systolic blood pressure |
|  | Neighbors of tumor resistance |
|  | Neighbors of blood glucose |
|  | Neighbors of ejection fraction |
|  | Neighbors of median survival |
|  | Neighbors of osteoclast number |
|  | Neighbors of cancer recurrence |
|  | Neighbors of T-cell count |
|  | Neighbors of antimetastatic activity |
|  | Neighbors of energy expenditure |
|  | Neighbors of radiosensitivity |
|  | Neighbors of left ventricular ejection fraction |
|  | Neighbors of cell permeability |
|  | Neighbors of pregnancy outcome |
|  | Neighbors of functional status |
|  | Neighbors of fertility |
|  | Neighbors of recurrence rate |
|  | Neighbors of pathological response |
|  | Neighbors of lymphocyte count |
|  | Neighbors of monocyte count |
|  | Neighbors of skin thickness |
|  | Neighbors of metabolic response |
|  | Neighbors of insulin level |
|  | Neighbors of comorbidity |
|  | Neighbors of carotid intima-media thickness |
|  | Neighbors of bone volume |
|  | Neighbors of fibroblast phenotype |
|  | Neighbors of fractional shortening |
|  | Neighbors of disease exacerbation |
|  | Neighbors of tumor progression |
|  | Neighbors of cancer progression |
|  | Neighbors of inflammatory status |
|  | Neighbors of overall survival |
|  | Neighbors of disease severity |
|  | Neighbors of tumor size |
|  | Neighbors of lesion size |
|  | Neighbors of mortality |
|  | Neighbors of survival rate |
|  | Neighbors of infarct size |
|  | Neighbors of survival time |
|  | Neighbors of functional recovery |
|  | Neighbors of insulin sensitivity |
|  | Neighbors of bacterial load |
|  | Neighbors of cancer survival |
|  | Neighbors of graft survival |
|  | Neighbors of therapeutic efficacy |
|  | Neighbors of clinical stage |
|  | Neighbors of lethality |
|  | Neighbors of tumor regression |
|  | Neighbors of recurrence-free survival |
|  | Neighbors of disease-free survival |
|  | Neighbors of tumor response |
|  | Neighbors of macrophage count |
|  | Neighbors of long-term survival |
|  | Neighbors of treatment outcome |
|  | Neighbors of bone mass |
|  | Neighbors of body weight |
|  | Neighbors of body weight gain |
|  | Neighbors of cardiovascular risk |
|  | Neighbors of microvessel density |
|  | Neighbors of tumor recurrence |
|  | Neighbors of tumor promotion |
|  | Neighbors of neutrophil count |
|  | Neighbors of glucose tolerance |
|  | Neighbors of morbidity |
|  | Neighbors of fibrosis progression |
|  | Neighbors of mortality rate |
|  | Neighbors of fat mass |
|  | Neighbors of exhaustion |
|  | Neighbors of bone mineral density |
|  | Neighbors of adverse outcome |
|  | Neighbors of lesion area |
|  | Neighbors of cell density |
|  | Neighbors of progression-free survival |
|  | Neighbors of apoptosis rate |
|  | Neighbors of early mortality |
|  | Neighbors of viral load |
|  | Neighbors of blood pressure |
|  | Neighbors of leukocyte count |
|  | Neighbors of vascular reactivity |
|  | Neighbors of vascular leakage |
|  | Neighbors of body mass index |
|  | Neighbors of vascular density |
|  | Neighbors of muscle mass |
|  | Neighbors of cancer incidence |
|  | Neighbors of systolic blood pressure |
|  | Neighbors of tumor resistance |
|  | Neighbors of blood glucose |
|  | Neighbors of ejection fraction |
|  | Neighbors of median survival |
|  | Neighbors of osteoclast number |
|  | Neighbors of cancer recurrence |
|  | Neighbors of T-cell count |
|  | Neighbors of antimetastatic activity |
|  | Neighbors of energy expenditure |
|  | Neighbors of radiosensitivity |
|  | Neighbors of left ventricular ejection fraction |
|  | Neighbors of cell permeability |
|  | Neighbors of pregnancy outcome |
|  | Neighbors of functional status |
|  | Neighbors of fertility |
|  | Neighbors of recurrence rate |
|  | Neighbors of pathological response |
|  | Neighbors of lymphocyte count |
|  | Neighbors of monocyte count |
|  | Neighbors of skin thickness |
|  | Neighbors of metabolic response |
|  | Neighbors of insulin level |
|  | Neighbors of comorbidity |
|  | Neighbors of carotid intima-media thickness |
|  | Neighbors of bone volume |
|  | Neighbors of fibroblast phenotype |
|  | Neighbors of fractional shortening |
|  | Neighbors of cell development |
|  | Neighbors of immune response |
|  | Neighbors of cell population |
|  | Neighbors of T-cell development |
|  | Neighbors of inflammatory response |
|  | Neighbors of wound healing |
|  | Neighbors of angiogenesis |
|  | Neighbors of innate immune response |
|  | Neighbors of cell function |
|  | Neighbors of macrophage activation |
|  | Neighbors of cell formation |
|  | Neighbors of adaptive immune response |
|  | Neighbors of osteoclast development |
|  | Neighbors of epithelial to mesenchymal transition |
|  | Neighbors of immunity |
|  | Neighbors of cell proliferative response |
|  | Neighbors of chemotaxis |
|  | Neighbors of dendritic cell differentiation |
|  | Neighbors of cell survival |
|  | Neighbors of osteoclast differentiation |
|  | Neighbors of cell phenotype |
|  | Neighbors of T-cell proliferation |
|  | Neighbors of tissue remodeling |
|  | Neighbors of cell adhesion |
|  | Neighbors of T-cell activation |
|  | Neighbors of endothelial cell proliferation |
|  | Neighbors of fibrogenesis |
|  | Neighbors of tumor growth |
|  | Neighbors of dendritic cell development |
|  | Neighbors of tissue repair |
|  | Neighbors of cell count |
|  | Neighbors of cell infiltration |
|  | Neighbors of cell interaction |
|  | Neighbors of SMC proliferation |
|  | Neighbors of lumen formation |
|  | Neighbors of endothelial cell migration |
|  | Neighbors of adipogenesis |
|  | Neighbors of lipid storage |
|  | Neighbors of cell damage |
|  | Neighbors of pregnancy |
|  | Neighbors of monocyte differentiation |
|  | Neighbors of aging |
|  | Neighbors of cell motility |
|  | Neighbors of bone resorption |
|  | Neighbors of monocyte migration |
|  | Neighbors of fibroblast proliferation |
|  | Neighbors of adipocyte differentiation |
|  | Neighbors of macrophage migration |
|  | Neighbors of microglial activation |
|  | Neighbors of neutrophil recruitment |
|  | Neighbors of regeneration |
|  | Neighbors of cell migration |
|  | Neighbors of cancer growth |
|  | Neighbors of stem cell proliferation |
|  | Neighbors of smooth muscle cell migration |
|  | Neighbors of hepatic regeneration |
|  | Neighbors of transendothelial migration |
|  | Neighbors of phagocytosis |
|  | Neighbors of immune system activation |
|  | Neighbors of leukocyte migration |
|  | Neighbors of leukocyte recruitment |
|  | Neighbors of osteoclast formation |
|  | Neighbors of monocyte recruitment |
|  | Neighbors of cell invasion |
|  | Neighbors of heart function |
|  | Neighbors of hemopoiesis |
|  | Neighbors of bone remodeling |
|  | Neighbors of ossification |
|  | Neighbors of T-cell function |
|  | Neighbors of immune system function |
|  | Neighbors of lung development |
|  | Neighbors of autophagy |
|  | Neighbors of monocyte adhesion |
|  | Neighbors of transcription activation |
|  | Neighbors of senescence |
|  | Neighbors of T-cell response |
|  | Neighbors of tumor immunity |
|  | Neighbors of hepatocyte proliferation |
|  | Neighbors of cell transdifferentiation |
|  | Neighbors of neuroprotection |
|  | Neighbors of stem cell differentiation |
|  | Neighbors of endothelial cell function |
|  | Neighbors of neuronal death |
|  | Neighbors of cell growth |
|  | Neighbors of ROS generation |
|  | Neighbors of epithelial cell proliferation |
|  | Neighbors of first trimester pregnancy |
|  | Neighbors of cancer cell growth |
|  | Neighbors of sensitization |
|  | Neighbors of hemato-encephalic barrier |
|  | Neighbors of macrophage function |
|  | Neighbors of cellular immune response |
|  | Neighbors of cellular senescence |
|  | Neighbors of colony formation |
|  | Neighbors of macrophage differentiation |
|  | Neighbors of cell differentiation |
|  | Neighbors of keratinocyte proliferation |
|  | Neighbors of stem cell migration |
|  | Neighbors of life span |
|  | Neighbors of cell homeostasis |
|  | Neighbors of psoriasis |
|  | Neighbors of inflammatory disease |
|  | Neighbors of rheumatoid arthritis |
|  | Neighbors of atherosclerosis |
|  | Neighbors of fibrosis |
|  | Neighbors of colitis |
|  | Neighbors of arthritis |
|  | Neighbors of inflammatory bowel disease |
|  | Neighbors of injury |
|  | Neighbors of atherogenesis |
|  | Neighbors of vascular remodeling |
|  | Neighbors of metastasis |
|  | Neighbors of carcinogenesis |
|  | Neighbors of multiple sclerosis |
|  | Neighbors of chronic inflammation |
|  | Neighbors of inflammation |
|  | Neighbors of tumor microenvironment |
|  | Neighbors of pathological angiogenesis |
|  | Neighbors of obesity |
|  | Neighbors of vascular disease |
|  | Neighbors of diabetes mellitus |
|  | Neighbors of asthma |
|  | Neighbors of pneumonia |
|  | Neighbors of hyperplasia |
|  | Neighbors of osteoarthritis |
|  | Neighbors of liver fibrosis |
|  | Neighbors of chronic obstructive pulmonary disease |
|  | Neighbors of myocardial infarction |
|  | Neighbors of leukocyte infiltration |
|  | Neighbors of reperfusion injury |
|  | Neighbors of airway inflammation |
|  | Neighbors of hepatitis |
|  | Neighbors of insulin resistance |
|  | Neighbors of infection |
|  | Neighbors of vascular injury |
|  | Neighbors of renal injury |
|  | Neighbors of preeclampsia |
|  | Neighbors of macrophage infiltration |
|  | Neighbors of liver injury |
|  | Neighbors of neuroinflammation |
|  | Neighbors of sepsis |
|  | Neighbors of death |
|  | Neighbors of type 1 diabetes |
|  | Neighbors of encephalomyelitis |
|  | Neighbors of autoimmune disease |
|  | Neighbors of ischemic stroke |
|  | Neighbors of dermatitis |
|  | Neighbors of neutrophil infiltration |
|  | Neighbors of heart disease |
|  | Neighbors of lung injury |
|  | Neighbors of immunopathology |
|  | Neighbors of systemic sclerosis |
|  | Neighbors of hepatocellular carcinoma |
|  | Neighbors of autoimmunity |
|  | Neighbors of acute lung injury |
|  | Neighbors of gastric cancer |
|  | Neighbors of atherosclerotic plaque |
|  | Neighbors of experimental autoimmune encephalomyelitis |
|  | Neighbors of colorectal cancer |
|  | Neighbors of cardiac remodeling |
|  | Neighbors of breast cancer |
|  | Neighbors of ischemia |
|  | Neighbors of systemic lupus erythematosus |
|  | Neighbors of melanoma |
|  | Neighbors of collagen-induced arthritis |
|  | Neighbors of cartilage degeneration |
|  | Neighbors of interstitial fibrosis |
|  | Neighbors of neoplasm invasion |
|  | Neighbors of kidney disease |
|  | Neighbors of heart failure |
|  | Neighbors of lung disease |
|  | Neighbors of pulmonary fibrosis |
|  | Neighbors of cardiovascular disease |
|  | Neighbors of stroke |
|  | Neighbors of cancer |
|  | Neighbors of cerebral ischemia |
|  | Neighbors of type 2 diabetes |
|  | Neighbors of neoplasm |
|  | Neighbors of endometriosis |
|  | Neighbors of neointima |
|  | Neighbors of vasculitis |
|  | Neighbors of atrophy |
|  | Neighbors of diet-induced obesity |
|  | Neighbors of respiratory hypersensitivity |
|  | Neighbors of myocardial fibrosis |
|  | Neighbors of synovitis |
|  | Neighbors of coronary artery disease |
|  | Neighbors of rupture |
|  | Neighbors of endothelial cell dysfunction |
|  | Neighbors of liver disease |
|  | Neighbors of prostate cancer |
|  | Neighbors of hypertrophy |
|  | Neighbors of osteolysis |
|  | Neighbors of ovarian cancer |
|  | Neighbors of pancreatic cancer |
|  | Neighbors of hypertension |
|  | Neighbors of intimal hyperplasia |
|  | Neighbors of allograft rejection |
|  | Neighbors of proteinuria |
|  | Neighbors of epithelial cell |
|  | Neighbors of keratinocyte |
|  | Neighbors of vascular smooth muscle cell |
|  | Neighbors of umbilical vein endothelial cell |
|  | Neighbors of monocyte |
|  | Neighbors of endometrial stromal cell |
|  | Neighbors of alveolar macrophage |
|  | Neighbors of synoviocyte |
|  | Neighbors of macrophage |
|  | Neighbors of adipocyte |
|  | Neighbors of activated monocyte |
|  | Neighbors of fibroblast |
|  | Neighbors of airway epithelial cell |
|  | Neighbors of PBMC |
|  | Neighbors of stromal cell |
|  | Neighbors of airway smooth muscle cell |
|  | Neighbors of osteoblast |
|  | Neighbors of pneumocyte |
|  | Neighbors of endometrium cell |
|  | Neighbors of microglia |
|  | Neighbors of trophoblast |
|  | Neighbors of intestine epithelium cell |
|  | Neighbors of mononuclear cell |
|  | Neighbors of hepatic stellate cell |
|  | Neighbors of hepatocyte |
|  | Neighbors of decidual cell |
|  | Neighbors of kidney mesangial cell |
|  | Neighbors of endothelial cell |
|  | Neighbors of dendritic cell |
|  | Neighbors of Kuppfer cell |
|  | Neighbors of kidney tubule cell |
|  | Neighbors of inflammatory cell |
|  | Neighbors of bone marrow macrophage |
|  | Neighbors of peritoneal macrophage |
|  | Neighbors of osteoclast |
|  | Neighbors of neutrophil |
|  | Neighbors of smooth muscle myocyte |
|  | Neighbors of astrocyte |
|  | Neighbors of bone marrow derived dendritic cell |
|  | Neighbors of activated T-cell |
|  | Neighbors of mononuclear phagocyte |
|  | Neighbors of capillary endothelial cell |
|  | Neighbors of immunocompetent cell |
|  | Neighbors of granulocyte |
|  | Neighbors of cardiac myocyte |
|  | Neighbors of cancer-associated fibroblast |
|  | Neighbors of myeloid cell |
|  | Neighbors of mesenchymal stroma cell |
|  | Neighbors of corneal epithelial cell |
|  | Neighbors of stem cell |
|  | Neighbors of tumor infiltrating macrophage |
|  | Neighbors of sinusoidal cell |
|  | Neighbors of B-cell |
|  | Neighbors of leukocyte |
|  | Neighbors of chondrocyte |
|  | Neighbors of insulin-secreting cell |
|  | Neighbors of mesenchymal cell |
|  | Neighbors of T-cell |
|  | Neighbors of M1 macrophage |
|  | Neighbors of mast cell |
|  | Neighbors of bone marrow stromal cell |
|  | Neighbors of spleen cell |
|  | Neighbors of brain capillary endothelial cell |
|  | Neighbors of neuroglia |
|  | Neighbors of thyroid epithelial cell |
|  | Neighbors of hematopoietic cell |
|  | Neighbors of adipose macrophage |
|  | Neighbors of mammary epithelial cell |
|  | Neighbors of conventional dendritic cell |
|  | Neighbors of pericyte |
|  | Neighbors of antigen-presenting cell |
|  | Neighbors of epidermis cell |
|  | Neighbors of bone marrow derived mast cell |
|  | Neighbors of phagocyte |
|  | Neighbors of kidney cell |
|  | Neighbors of syncytiotrophoblast |
|  | Neighbors of lymphocyte |
|  | Neighbors of Langerhans cell |
|  | Neighbors of decidual natural killer cell |
|  | Neighbors of thymic epithelial cell |
|  | Neighbors of brain cell |
|  | Neighbors of M2 macrophage |
|  | Neighbors of peritoneum cell |
|  | Neighbors of lymphoid cell |
|  | Neighbors of blood cell |
|  | Neighbors of skin cell |
|  | Neighbors of luteal cell |
|  | Neighbors of peripheral lymphocyte |
|  | Neighbors of activated B-cell |
|  | Neighbors of lymph node cell |
|  | Neighbors of lung |
|  | Neighbors of liver |
|  | Neighbors of kidney |
|  | Neighbors of skin |
|  | Neighbors of intestine |
|  | Neighbors of lymph node |
|  | Neighbors of colon |
|  | Neighbors of airway |
|  | Neighbors of blood vessel |
|  | Neighbors of heart |
|  | Neighbors of aorta |
|  | Neighbors of gastrointestinal tract |
|  | Neighbors of microvessel |
|  | Neighbors of bone |
|  | Neighbors of placenta |
|  | Neighbors of synovial membrane |
|  | Neighbors of uterus |
|  | Neighbors of periodontium |
|  | Neighbors of retina |
|  | Neighbors of brain |
|  | Neighbors of breast |
|  | Neighbors of blood vessel wall |
|  | Neighbors of stomach |
|  | Neighbors of islets of Langerhans |
|  | Neighbors of joint |
|  | Neighbors of artery |
|  | Neighbors of pancreas |
|  | Neighbors of prostate |
|  | Neighbors of central nervous system |
|  | Neighbors of kidney glomerulus |
|  | Neighbors of lymphatic system |
|  | Neighbors of colorectal region |
|  | Neighbors of decidua |
|  | Neighbors of immune system |
|  | Neighbors of spleen |
|  | Neighbors of peritoneum |
|  | Neighbors of alveolus |
|  | Neighbors of left ventricle |
|  | Neighbors of uterine cervix |
|  | Neighbors of bronchus |
|  | Neighbors of glands |
|  | Neighbors of nose |
|  | Neighbors of eye |
|  | Neighbors of cornea |
|  | Neighbors of lymph node of mesentery |
|  | Neighbors of vagina |
|  | Neighbors of thymus gland |
|  | Neighbors of cerebral cortex |
|  | Neighbors of myelin sheath |
|  | Neighbors of bladder |
|  | Neighbors of draining lymph node |
|  | Neighbors of nervous system |
|  | Neighbors of mesentery |
|  | Neighbors of nasopharynx |
|  | Neighbors of cervical lymph node group |
|  | Neighbors of salivary gland |
|  | Neighbors of trachea |
|  | Neighbors of nerve |
|  | Neighbors of ileum |
|  | Neighbors of endocrine system |
|  | Neighbors of small intestine |
|  | Neighbors of aggregated lymphoid follicle of small intestine |
|  | Neighbors of globus pallidus |
|  | Neighbors of knee joint |
|  | Neighbors of mediastinal lymph node group |
|  | Neighbors of reproductive system |
|  | Neighbors of pleura |
|  | Neighbors of NF-kB family |
|  | Neighbors of oxidized LDL |
|  | Neighbors of JNK |
|  | Neighbors of IL1 family |
|  | Neighbors of PPAR |
|  | Neighbors of Jun/Fos |
|  | Neighbors of PKC |
|  | Neighbors of PI3K |
|  | Neighbors of STAT family |
|  | Neighbors of mitogen-activated protein kinase |
|  | Neighbors of IFNAR ligand |
|  | Neighbors of transforming growth factor |
|  | Neighbors of TLR |
|  | Neighbors of cytokine |
|  | Neighbors of interferon |
|  | Neighbors of interleukin |
|  | Neighbors of JAK |
|  | Neighbors of MEK1/2 |
|  | Neighbors of Ras GTPase |
|  | Neighbors of ERK1/2 |
|  | Neighbors of growth factor |
|  | Neighbors of PKA |
|  | Neighbors of ovalbumin |
|  | Neighbors of NF-AT family |
|  | Neighbors of beta adrenoceptor |
|  | Neighbors of chorionic gonadotropin |
|  | Neighbors of IKBK |
|  | Neighbors of adipokine |
|  | Neighbors of protein tyrosine kinase |
|  | Neighbors of NAD(P)H oxidase |
|  | Neighbors of IgG |
|  | Neighbors of SMAD subfamily |
|  | Neighbors of AMPK |
|  | Neighbors of LXR |
|  | Neighbors of HDL |
|  | Neighbors of p38 |
|  | Neighbors of inflammatory cytokine |
|  | Neighbors of IL23 |
|  | Neighbors of estrogen receptor |
|  | Neighbors of T-cell receptor |
|  | Neighbors of histone deacetylase |
|  | Neighbors of PDGF-BB |
|  | Neighbors of IL1R |
|  | Neighbors of GPCR |
|  | Neighbors of IL12 |
|  | Neighbors of Notch |
|  | Neighbors of prostaglandin-endoperoxide synthase |
|  | Neighbors of heat shock protein 90 |
|  | Neighbors of lectin |
|  | Neighbors of fibrinogen |
|  | Neighbors of aldehyde reductase |
|  | Neighbors of mitogen-activated protein kinase kinase |
|  | Neighbors of interleukin-35 |
|  | Neighbors of Ang II receptor |
|  | Neighbors of endothelin |
|  | Neighbors of superoxide dismutase |
|  | Neighbors of non-coding RNA |
|  | Neighbors of STAT5 |
|  | Neighbors of alpha2 adrenoceptor |
|  | Neighbors of PP2A |
|  | Neighbors of retinoid-X receptor subfamily |
|  | Neighbors of WNT |
|  | Neighbors of gonadotropins |
|  | Neighbors of immunoglobulin |
|  | Neighbors of PP2B |
|  | Neighbors of phospholipase |
|  | Neighbors of AHR subfamily |
|  | Neighbors of CD3 complex |
|  | Neighbors of relaxin |
|  | Neighbors of TLR1/2 heterodimer |
|  | Neighbors of TORC1 |
|  | Neighbors of angiotensin receptor |
|  | Neighbors of RA receptor |
|  | Neighbors of histone |
|  | Neighbors of Rho kinase |
|  | Neighbors of neuropeptides |
|  | Neighbors of adrenoceptor |
|  | Neighbors of phosphodiesterase |
|  | Neighbors of phosphoinositide phospholipase C |
|  | comon ps\_pos, pprarg\_neg targets |
|  | Model of PPARG signaling in psoriasis |
|  | PPARG negative regulators and targets |
|  | Model of PPARG related pathways in psoriasis (short version) |
|  | PPARs and anxiety common targets |
|  | Amygdaloid expressed genes |
|  | PPARs and neuroinflamation |
|  | New Pathway (3) |
|  | dsRNA induces innate immunity and INFs in alveolar cells |
|  | IFN signaling in alveolar cells |
|  | Overview. Diseases of the skin (chapter 11) |
|  | Overview. Diseases of the respiratory (chapter 9) |
|  | Overview. Diseases of the genitourinary system (chapter 13) |
|  | Overview. Infectious and parasitic diseases (chapter 2) |
|  | 1\_Differentiation of psoriatic T cells |
|  | 1\_The onset of atopic dermatitis |
|  | 1\_Defects in response to pathogens in the gut promotes inflammation in Crohn's disease |
|  | 3\_Autophagy dysfunction in Crohn's disease |
|  | 4\_2\_Genes with mutations currently associated with ulcerative colitis |
|  | 2\_Intestine epithelial cells drive immunological responses in ulcerative colitis |
|  | 4\_1\_Polymorphisms associated with inflammatory bowel diseases |
|  | 1\_CD4+ T cell and epithelial cell response in celiac disease |
|  | 2\_CD8+ T-cell response and mucosal damage in celiac disease |
|  | 2\_High levels of hepcidin block iron absorption in the gut |
|  | 3\_Impairment of iron release from splenic macrophages |
|  | 4\_Inflammatory mediators inhibit GATA signaling in erythropoiesis |
|  | 1\_Antigen-presenting cells promote islet dysfunction and immune system activation in T1D |
|  | 3\_Islet beta-cell destruction in T1D |
|  | 2\_Defective tolerance of autoreactive T-cell in T1D |
|  | drug-target pairs direct regulation |
|  | drug-target moltransport |
|  | New Combined Pathway |
|  | Differentiation of psoriatic T cells |

---

|  |  |
| --- | --- |
| Group | Genes with Mutations Associated with Hashimoto's Thyroiditis |
|  | Genes with Mutations Associated with Graves Ophthalmopathy |
|  | Genes with Mutations Associated with Multiple Sclerosis |

---

|  |  |
| --- | --- |
| Source | curated |
|  | GO component |
|  | ResNet |

---

|  |  |
| --- | --- |
| MedScan ID | 3458 |

---

|  |  |
| --- | --- |
| LocusLink ID | 3458 |
|  | 15978 |
|  | 25712 |
|  | 281237 |
|  | 405790 |

---

|  |  |
| --- | --- |
| Alias | ifng |
|  | gamma IFN |
|  | interferon type II |
|  | interferon gamma 2 |
|  | gamma interferon 2 |
|  | IFN-g |
|  | Immune interferon |
|  | interferon gamma |
|  | human immune interferon |
|  | IFNgamma |
|  | gamma interferon |
|  | IFNG2 |
|  | ING\_HUMAN |
|  | oh 6000 |
|  | imunomax gamma |
|  | interferon-gamma receptor ligand |
|  | interferon 2 |
|  | interferon-g |
|  | gamma 2 interferon |
|  | IFG |
|  | IFI |
|  | IFN-gamma |
|  | interferon, gamma |
|  | IFN gamma |
|  | Interferon gamma precursor |
|  | ING |
|  | CH211-266A5.7 |
|  | INF-G |
|  | chromosome |
|  | gamma |
|  | ifng1-2 |
|  | interferon |
|  | interferon, gamma 1-2 |
|  | scaffold |

---

|  |  |
| --- | --- |
| GO ID | 0005125 |
|  | 0005133 |
|  | 0007259 |
|  | 0002250 |
|  | 0006915 |
|  | 0048143 |
|  | 0007050 |
|  | 0007166 |
|  | 0051607 |
|  | 0097191 |
|  | 0006959 |
|  | 0060333 |
|  | 0035722 |
|  | 0001774 |
|  | 1900222 |
|  | 0030857 |
|  | 0010629 |
|  | 0032700 |
|  | 0048662 |
|  | 1902948 |
|  | 0000122 |
|  | 0045892 |
|  | 0150076 |
|  | 0032834 |
|  | 0045348 |
|  | 1904783 |
|  | 1902004 |
|  | 0010508 |
|  | 0060559 |
|  | 0008284 |
|  | 1901857 |
|  | 0045080 |
|  | 1904798 |
|  | 0010634 |
|  | 1903543 |
|  | 1904440 |
|  | 0060550 |
|  | 0060552 |
|  | 0010628 |
|  | 0045821 |
|  | 0050725 |
|  | 0032735 |
|  | 0032747 |
|  | 0051712 |
|  | 0051044 |
|  | 0050769 |
|  | 1901216 |
|  | 0045429 |
|  | 0051770 |
|  | 0051173 |
|  | 0045672 |
|  | 0033141 |
|  | 0050766 |
|  | 0031334 |
|  | 0090312 |
|  | 0042307 |
|  | 1903078 |
|  | 0001934 |
|  | 0071902 |
|  | 0010870 |
|  | 0034393 |
|  | 2000309 |
|  | 0042535 |
|  | 1904469 |
|  | 0042531 |
|  | 0060557 |
|  | 0040008 |
|  | 0050796 |
|  | 0060334 |
|  | 0010835 |
|  | 0045589 |
|  | 0009615 |
|  | 0005576 |
|  | 0005615 |
|  | 0002302 |
|  | 0050852 |
|  | 0019882 |
|  | 0071351 |
|  | 0071222 |
|  | 0042742 |
|  | 0042832 |
|  | 0030968 |
|  | 0006955 |
|  | 0006925 |
|  | 0008285 |
|  | 0048147 |
|  | 0072125 |
|  | 0044130 |
|  | 0044146 |
|  | 0031642 |
|  | 0045671 |
|  | 0001781 |
|  | 0030593 |
|  | 0044794 |
|  | 0042102 |
|  | 0043065 |
|  | 0045785 |
|  | 0050718 |
|  | 0045084 |
|  | 0045410 |
|  | 0048304 |
|  | 0045666 |
|  | 0032224 |
|  | 0045944 |
|  | 0045893 |
|  | 0032760 |
|  | 0050691 |
|  | 0060251 |
|  | 2000345 |
|  | 0050776 |
|  | 0098908 |
|  | 0002026 |
|  | 0006355 |
|  | 0050954 |
|  | 0005737 |
|  | 0009897 |
|  | 0043005 |
|  | 0043204 |
|  | 0042493 |
|  | 0005622 |
|  | 0000060 |
|  | 0010469 |
|  | 0033160 |
|  | 0005623 |
|  | 0019221 |
|  | 0002053 |
|  | 0006928 |
|  | 0003340 |
|  | 0042511 |
|  | 0090004 |
|  | 0072308 |
|  | 0010553 |
|  | 0042508 |
|  | 0010552 |
|  | 0045449 |
|  | 0001558 |
|  | 0042830 |
|  | 0006952 |
|  | 0016563 |
|  | 0007267 |

---

|  |  |
| --- | --- |
| KEGG ID | rno:25712 |
|  | hsa:3458 |
|  | mmu:15978 |

---

|  |  |
| --- | --- |
| MedLine Reference | 8097205 |
|  | 17534845 |
|  | 17351619 |
|  | 15585884 |
|  | 2504704 |
|  | 10413362 |
|  | 16118315 |
|  | 16966429 |
|  | 14764711 |
|  | 11197690 |
|  | 15388256 |
|  | 16148108 |
|  | 11921420 |
|  | 15240659 |
|  | 11984595 |
|  | 16484229 |
|  | 17585067 |
|  | 1572101 |
|  | 10725727 |
|  | 16369024 |
|  | 11774563 |
|  | 8118869 |
|  | 15778393 |
|  | 15681246 |
|  | 15652446 |
|  | 16280381 |
|  | 7556951 |
|  | 12466851 |
|  | 14732350 |
|  | 16323704 |
|  | 17339356 |
|  | 7903095 |
|  | 16564704 |
|  | 17412309 |
|  | 1708818 |
|  | 17697357 |
|  | 12851716 |
|  | 16224059 |
|  | 16678358 |
|  | 14764734 |
|  | 11104801 |
|  | 15967822 |
|  | 12548511 |
|  | 17339353 |
|  | 10553050 |
|  | 11782537 |
|  | 17304101 |
|  | 15967825 |
|  | 7595475 |
|  | 14733929 |
|  | 17154717 |
|  | 17304110 |
|  | 16103182 |
|  | 15039223 |
|  | 14962095 |
|  | 16024776 |
|  | 15778370 |
|  | 10358171 |
|  | 17082625 |
|  | 12239181 |
|  | 16034083 |
|  | 11390498 |
|  | 14742442 |
|  | 17244673 |
|  | 17292338 |
|  | 10639149 |
|  | 14764721 |
|  | 15297269 |
|  | 15604419 |
|  | 15604417 |
|  | 11141333 |
|  | 12928400 |
|  | 9164971 |
|  | 15778381 |
|  | 12147624 |
|  | 12496185 |
|  | 15985639 |
|  | 12928402 |
|  | 1422836 |
|  | 17208926 |
|  | 15578690 |
|  | 9485200 |
|  | 16102751 |
|  | 12370378 |
|  | 12442336 |
|  | 11823506 |
|  | 9381654 |
|  | 9590262 |
|  | 15935691 |
|  | 15367434 |
|  | 8432988 |
|  | 12933895 |
|  | 16201318 |
|  | 12471131 |
|  | 12471132 |
|  | 12042661 |
|  | 11823513 |
|  | 12471139 |
|  | 15509523 |
|  | 11687972 |
|  | 11823519 |
|  | 9070650 |
|  | 9971752 |
|  | 16766656 |
|  | 15499631 |
|  | 12645529 |
|  | 12972478 |
|  | 12392275 |
|  | 10375558 |
|  | 16906223 |
|  | 7601474 |
|  | 11290561 |
|  | 12401479 |
|  | 17307940 |
|  | 16966404 |
|  | 8006599 |
|  | 12244183 |
|  | 17307945 |
|  | 15128787 |
|  | 12850812 |
|  | 9288735 |
|  | 12850811 |
|  | 12850810 |
|  | 14976252 |
|  | 15100264 |
|  | 8890206 |
|  | 12370350 |
|  | 10072260 |
|  | 11466347 |
|  | 16266858 |
|  | 12925700 |
|  | 11042159 |
|  | 11728336 |
|  | 10233748 |
|  | 7532600 |
|  | 15531883 |
|  | 9239401 |
|  | 17038663 |
|  | 16150647 |
|  | 7884320 |
|  | 11673544 |
|  | 16680145 |
|  | 15466391 |
|  | 16790816 |
|  | 16966412 |
|  | 7906942 |
|  | 11390451 |
|  | 15568025 |
|  | 16507895 |
|  | 8071611 |
|  | 16280321 |
|  | 11751951 |
|  | 9590248 |
|  | 10875919 |
|  | 14704034 |
|  | 12859963 |
|  | 16553809 |
|  | 17334371 |
|  | 15611472 |
|  | 16257975 |
|  | 11673566 |
|  | 11057672 |
|  | 14502280 |
|  | 10477596 |
|  | 15536146 |
|  | 15044949 |
|  | 9820535 |
|  | 15654515 |
|  | 7676639 |
|  | 16614074 |
|  | 8760410 |
|  | 12907450 |
|  | 8100556 |
|  | 17278885 |
|  | 9802977 |
|  | 11908942 |
|  | 15809350 |
|  | 16421220 |
|  | 14753294 |
|  | 7974717 |
|  | 15068623 |
|  | 16014561 |
|  | 11937520 |
|  | 16914507 |
|  | 16897354 |
|  | 14978081 |
|  | 15555926 |
|  | 17556594 |
|  | 10072077 |
|  | 10640742 |
|  | 9344654 |
|  | 17566759 |
|  | 8545890 |
|  | 17229673 |
|  | 7826951 |
|  | 15115662 |
|  | 14500652 |
|  | 17037575 |
|  | 16026997 |
|  | 8752931 |
|  | 8960823 |
|  | 1924386 |
|  | 14605445 |
|  | 16921135 |
|  | 9653090 |
|  | 15539155 |
|  | 12102718 |
|  | 11067886 |
|  | 17157914 |
|  | 9892605 |
|  | 15521020 |
|  | 17548597 |
|  | 14500669 |
|  | 16085646 |
|  | 12165484 |
|  | 14684702 |
|  | 17178781 |
|  | 15383459 |
|  | 15804285 |
|  | 16724074 |
|  | 15374002 |
|  | 16214174 |
|  | 17110450 |
|  | 15804287 |
|  | 17202360 |
|  | 15628695 |
|  | 15145690 |
|  | 12192641 |
|  | 17477820 |
|  | 15723067 |
|  | 15383460 |
|  | 9670937 |
|  | 9935167 |
|  | 12740040 |
|  | 15797996 |
|  | 15681825 |
|  | 8911153 |
|  | 16357940 |
|  | 15182335 |
|  | 12020956 |
|  | 11412311 |
|  | 12719555 |
|  | 15037019 |
|  | 17093503 |
|  | 11489982 |
|  | 12438448 |
|  | 17395783 |
|  | 7507352 |
|  | 12707360 |
|  | 17565340 |
|  | 16002714 |
|  | 11714812 |
|  | 12653164 |
|  | 9548487 |
|  | 15343387 |
|  | 15710386 |
|  | 16428751 |
|  | 8011213 |
|  | 15580300 |
|  | 12555666 |
|  | 16478776 |
|  | 16002721 |
|  | 11087156 |
|  | 1359872 |
|  | 12198617 |
|  | 14742242 |
|  | 8778035 |
|  | 11348452 |
|  | 7908325 |
|  | 9548493 |
|  | 8456300 |
|  | 9570555 |
|  | 11576226 |
|  | 15265903 |
|  | 10078962 |
|  | 16467877 |
|  | 15944319 |
|  | 14991597 |
|  | 11947926 |
|  | 15694390 |
|  | 8619916 |
|  | 8200040 |
|  | 15611248 |
|  | 9824514 |
|  | 7547684 |
|  | 17051153 |
|  | 12477932 |
|  | 15983045 |
|  | 12221108 |
|  | 16849446 |
|  | 17101606 |
|  | 17059581 |
|  | 15585862 |
|  | 12802010 |
|  | 7822804 |
|  | 16284650 |
|  | 15550390 |
|  | 15778340 |
|  | 12486605 |
|  | 9435308 |
|  | 9765476 |
|  | 1905983 |
|  | 15327519 |
|  | 1315358 |
|  | 12105273 |
|  | 11920321 |
|  | 12707353 |
|  | 12751024 |
|  | 8990384 |
|  | 8990382 |
|  | 15345584 |
|  | 15316224 |
|  | 15800938 |
|  | 16914208 |
|  | 9914220 |
|  | 11489988 |
|  | 17185357 |
|  | 8454850 |
|  | 11714835 |
|  | 9820529 |
|  | 16428767 |
|  | 15265921 |
|  | 16216888 |
|  | 10458761 |
|  | 11342667 |
|  | 15921521 |
|  | 11477543 |
|  | 12882831 |
|  | 8833235 |
|  | 9550369 |
|  | 8037746 |
|  | 15356138 |
|  | 8972906 |
|  | 11349033 |
|  | 12813035 |
|  | 7617032 |
|  | 15356141 |
|  | 14634104 |
|  | 16127458 |
|  | 16949558 |
|  | 10623823 |
|  | 12648490 |
|  | 11023473 |
|  | 15596864 |
|  | 16108727 |
|  | 9922312 |
|  | 11830474 |
|  | 11830476 |
|  | 17070076 |
|  | 11350823 |
|  | 7970703 |
|  | 11123351 |
|  | 17158904 |
|  | 8892621 |
|  | 15624893 |
|  | 16126843 |
|  | 12191570 |
|  | 15845473 |
|  | 10490099 |
|  | 12704788 |
|  | 16172135 |
|  | 10452977 |
|  | 9697983 |
|  | 12533683 |
|  | 15879081 |
|  | 17006334 |
|  | 14656873 |
|  | 16883061 |
|  | 10452966 |
|  | 17331636 |
|  | 12060463 |
|  | 14633605 |
|  | 12006410 |
|  | 12377943 |
|  | 11046015 |
|  | 12039912 |
|  | 12960156 |
|  | 8892638 |
|  | 10908669 |
|  | 17033822 |
|  | 11714750 |
|  | 15512964 |
|  | 12370192 |
|  | 10975822 |
|  | 15829292 |
|  | 10377194 |
|  | 16331625 |
|  | 8884266 |
|  | 14593499 |
|  | 16293599 |
|  | 15322002 |
|  | 8065455 |
|  | 10438942 |
|  | 9463404 |
|  | 12479817 |
|  | 7541825 |
|  | 15743488 |
|  | 15322007 |
|  | 15942146 |
|  | 12854077 |
|  | 15501397 |
|  | 12953847 |
|  | 12228294 |
|  | 3079789 |
|  | 9169855 |
|  | 15122910 |
|  | 14634140 |
|  | 14734771 |
|  | 9857043 |
|  | 15265878 |
|  | 8598493 |
|  | 10201893 |
|  | 16687505 |
|  | 11964313 |
|  | 14734769 |
|  | 12531694 |
|  | 11937574 |
|  | 11972612 |
|  | 15491990 |
|  | 17194566 |
|  | 14558082 |
|  | 15351034 |
|  | 16200070 |
|  | 17616703 |
|  | 16386911 |
|  | 16002423 |
|  | 10558953 |
|  | 15324937 |
|  | 6176945 |
|  | 9886418 |
|  | 15265886 |
|  | 17262717 |
|  | 12732663 |
|  | 10963809 |
|  | 15307168 |
|  | 10679111 |
|  | 14734753 |
|  | 15307169 |
|  | 2494257 |
|  | 12183572 |
|  | 11781185 |
|  | 10679118 |
|  | 8133052 |
|  | 16210597 |
|  | 11830478 |
|  | 16210591 |
|  | 10756011 |
|  | 15314695 |
|  | 17211638 |
|  | 14983028 |
|  | 15390286 |
|  | 15284232 |
|  | 12242445 |
|  | 11781187 |
|  | 12668667 |
|  | 9780277 |
|  | 16574426 |
|  | 12451269 |
|  | 12626579 |
|  | 15905622 |
|  | 12612906 |
|  | 11781192 |
|  | 10712678 |
|  | 9802888 |
|  | 12060750 |
|  | 9794424 |
|  | 17336935 |
|  | 17136124 |
|  | 2860101 |
|  | 10438967 |
|  | 17148586 |
|  | 9881967 |
|  | 9878080 |
|  | 15765122 |
|  | 8667616 |
|  | 10590269 |
|  | 15579433 |
|  | 14734739 |
|  | 10631568 |
|  | 10860730 |
|  | 16310045 |
|  | 8671675 |
|  | 17105733 |
|  | 10190695 |
|  | 15585559 |
|  | 8982782 |
|  | 15100252 |
|  | 15871905 |
|  | 15292059 |
|  | 16651614 |
|  | 15613333 |
|  | 8307620 |
|  | 12366695 |
|  | 11986281 |
|  | 17546034 |
|  | 15361241 |
|  | 12127799 |
|  | 16129707 |
|  | 7561104 |
|  | 7916324 |
|  | 15265425 |
|  | 16390939 |
|  | 12149429 |
|  | 16116201 |
|  | 17003472 |
|  | 16394017 |
|  | 11082062 |
|  | 12097431 |
|  | 6180322 |
|  | 11986277 |
|  | 15471952 |
|  | 16884913 |
|  | 15166131 |
|  | 15067054 |
|  | 17296747 |
|  | 14999686 |
|  | 8871613 |
|  | 14707048 |
|  | 11371356 |
|  | 9922399 |
|  | 16714546 |
|  | 17521731 |
|  | 16690980 |
|  | 15067057 |
|  | 17195845 |
|  | 16386594 |
|  | 16385625 |
|  | 17157804 |
|  | 8871615 |
|  | 7927491 |
|  | 9933454 |
|  | 16299343 |
|  | 10973283 |
|  | 12207333 |
|  | 15114672 |
|  | 17003484 |
|  | 16364177 |
|  | 3109913 |
|  | 12875978 |
|  | 12370382 |
|  | 15126416 |
|  | 12370385 |
|  | 12370386 |
|  | 11896209 |
|  | 15855642 |
|  | 17005003 |
|  | 16339569 |
|  | 16339567 |
|  | 15878863 |
|  | 15908417 |
|  | 14688303 |
|  | 10570301 |
|  | 11923871 |
|  | 11585387 |
|  | 1640172 |
|  | 17052246 |
|  | 9531602 |
|  | 10438928 |
|  | 16670308 |
|  | 15557182 |
|  | 7935058 |
|  | 12695304 |
|  | 11739503 |
|  | 12429523 |
|  | 11986256 |
|  | 15816837 |
|  | 16843267 |
|  | 8530075 |
|  | 17021766 |
|  | 16714568 |
|  | 16239557 |
|  | 10047537 |
|  | 12097414 |
|  | 16339555 |
|  | 16177352 |
|  | 8974835 |
|  | 16464236 |
|  | 7621071 |
|  | 10780549 |
|  | 17495004 |
|  | 14707092 |
|  | 14706232 |
|  | 16472593 |
|  | 11876761 |
|  | 11831440 |
|  | 10333049 |
|  | 17045255 |
|  | 12244202 |
|  | 15623791 |
|  | 17314060 |
|  | 11865054 |
|  | 16051062 |
|  | 15081598 |
|  | 17641033 |
|  | 11786644 |
|  | 15322214 |
|  | 17238853 |
|  | 15880135 |
|  | 7558008 |
|  | 16434471 |
|  | 11986939 |
|  | 17641021 |
|  | 7869053 |
|  | 14607827 |
|  | 11936954 |
|  | 15322205 |
|  | 16148152 |
|  | 17510508 |
|  | 11857344 |
|  | 12875993 |
|  | 12750172 |
|  | 12213961 |
|  | 3221389 |
|  | 12162871 |
|  | 6329718 |
|  | 12857673 |
|  | 17121582 |
|  | 11092553 |
|  | 1525157 |
|  | 16148138 |
|  | 14675398 |
|  | 17641061 |
|  | 12162877 |
|  | 12162878 |
|  | 12162874 |
|  | 15162834 |
|  | 9175833 |
|  | 16923852 |
|  | 15066699 |
|  | 12626771 |
|  | 12011029 |
|  | 16180247 |
|  | 16839357 |
|  | 16116224 |
|  | 14511565 |
|  | 7587386 |
|  | 12193724 |
|  | 16636173 |
|  | 7504064 |
|  | 16195537 |
|  | 14747539 |
|  | 12193725 |
|  | 10228024 |
|  | 15661939 |
|  | 15213124 |
|  | 14690454 |
|  | 11309656 |
|  | 14688135 |
|  | 16095488 |
|  | 16393991 |
|  | 17307864 |
|  | 17215375 |
|  | 15522880 |
|  | 10880532 |
|  | 11854218 |
|  | 12048245 |
|  | 12738388 |
|  | 10880533 |
|  | 15470062 |
|  | 7561088 |
|  | 15051500 |
|  | 16251408 |
|  | 8921430 |
|  | 16920960 |
|  | 12218156 |
|  | 12438348 |
|  | 17392368 |
|  | 11953317 |
|  | 9973424 |
|  | 15086382 |
|  | 1981053 |
|  | 12538662 |
|  | 11312140 |
|  | 7722464 |
|  | 12122108 |
|  | 16516165 |
|  | 16423040 |
|  | 11217851 |
|  | 16212914 |
|  | 7606797 |
|  | 16943259 |
|  | 15900487 |
|  | 12218120 |
|  | 16237070 |
|  | 7705395 |
|  | 17207783 |
|  | 12517946 |
|  | 14691261 |
|  | 15501793 |
|  | 7948748 |
|  | 11117749 |
|  | 16354185 |
|  | 12709397 |
|  | 12218136 |
|  | 7594453 |
|  | 12453468 |
|  | 15210741 |
|  | 11940252 |
|  | 11559791 |
|  | 16204617 |
|  | 12609974 |
|  | 1975791 |
|  | 14742542 |
|  | 12650767 |
|  | 16616105 |
|  | 12819637 |
|  | 11854210 |
|  | 8207233 |
|  | 15511228 |
|  | 12226082 |
|  | 11161447 |
|  | 15919906 |
|  | 16818748 |
|  | 17028199 |
|  | 15829401 |
|  | 9708573 |
|  | 16944293 |
|  | 11698474 |
|  | 16237054 |
|  | 16237051 |
|  | 12917425 |
|  | 16301680 |
|  | 12067206 |
|  | 16696900 |
|  | 11777610 |
|  | 9858522 |
|  | 16718821 |
|  | 17227821 |
|  | 14661818 |
|  | 15153506 |
|  | 15848040 |
|  | 15153508 |
|  | 16115026 |
|  | 16920922 |
|  | 8757628 |
|  | 17267208 |
|  | 10749577 |
|  | 12011077 |
|  | 17363736 |
|  | 16289836 |
|  | 7813776 |
|  | 8045595 |
|  | 7722423 |
|  | 17674321 |
|  | 16495808 |
|  | 15254597 |
|  | 10072551 |
|  | 11966872 |
|  | 15780879 |
|  | 14688108 |
|  | 9344502 |
|  | 12438388 |
|  | 11745345 |
|  | 16081786 |
|  | 14977944 |
|  | 16979605 |
|  | 12170266 |
|  | 6433489 |
|  | 12225958 |
|  | 8679287 |
|  | 15919929 |
|  | 14607900 |
|  | 16227271 |
|  | 1649220 |
|  | 12639998 |
|  | 11796707 |
|  | 16237056 |
|  | 16920936 |
|  | 12223098 |
|  | 15725351 |
|  | 12296868 |
|  | 16769892 |
|  | 17038535 |
|  | 9490698 |
|  | 16670325 |
|  | 15153522 |
|  | 17182600 |
|  | 10982767 |
|  | 12469117 |
|  | 14978106 |
|  | 12609526 |
|  | 15060019 |
|  | 17467814 |
|  | 10712011 |
|  | 17013806 |
|  | 17475277 |
|  | 12581491 |
|  | 10793093 |
|  | 16002150 |
|  | 16123425 |
|  | 8229991 |
|  | 14977926 |
|  | 17130300 |
|  | 15215891 |
|  | 17142760 |
|  | 15629884 |
|  | 2532365 |
|  | 10944420 |
|  | 12581489 |
|  | 10823854 |
|  | 17035315 |
|  | 17698562 |
|  | 15488732 |
|  | 2111015 |
|  | 16275384 |
|  | 15492127 |
|  | 15708587 |
|  | 16299306 |
|  | 15240714 |
|  | 14601649 |
|  | 8486363 |
|  | 15240719 |
|  | 8835541 |
|  | 15385479 |
|  | 14978132 |
|  | 10231581 |
|  | 15908180 |
|  | 14499500 |
|  | 8245795 |
|  | 8258693 |
|  | 17015711 |
|  | 12640294 |
|  | 16299319 |
|  | 12444137 |
|  | 16034116 |
|  | 11141495 |
|  | 12209087 |
|  | 9605173 |
|  | 11085872 |
|  | 16367922 |
|  | 14730360 |
|  | 7849395 |
|  | 8138038 |
|  | 12594832 |
|  | 15304658 |
|  | 16311601 |
|  | 10586042 |
|  | 8747928 |
|  | 12594839 |
|  | 8570653 |
|  | 11466402 |
|  | 15860458 |
|  | 15187132 |
|  | 16318585 |
|  | 16978068 |
|  | 9697730 |
|  | 12117934 |
|  | 11466408 |
|  | 1409609 |
|  | 17159910 |
|  | 11841489 |
|  | 12542496 |
|  | 17523872 |
|  | 16621996 |
|  | 12153990 |
|  | 16785527 |
|  | 15805249 |
|  | 10075980 |
|  | 15489334 |
|  | 15462469 |
|  | 11337387 |
|  | 10411532 |
|  | 14558106 |
|  | 16177085 |
|  | 17635807 |
|  | 12050152 |
|  | 9794412 |
|  | 11911824 |
|  | 10358203 |
|  | 15240709 |
|  | 15950283 |
|  | 16521127 |
|  | 15556691 |
|  | 15347683 |
|  | 9480982 |
|  | 11911827 |
|  | 12925694 |
|  | 16785519 |
|  | 16621981 |
|  | 8228624 |
|  | 11770039 |
|  | 15870936 |
|  | 17438029 |
|  | 15187119 |
|  | 9464804 |
|  | 12239295 |
|  | 12108917 |
|  | 9317148 |
|  | 15187113 |
|  | 15721283 |
|  | 17242058 |
|  | 11954833 |
|  | 9120292 |
|  | 14976609 |
|  | 12887272 |
|  | 15718915 |
|  | 12023376 |
|  | 16981183 |
|  | 10754318 |
|  | 15688349 |
|  | 9834083 |
|  | 10878379 |
|  | 8220433 |
|  | 12473063 |
|  | 8814290 |
|  | 8527411 |
|  | 16098562 |
|  | 10775602 |
|  | 11318565 |
|  | 11861290 |
|  | 15778222 |
|  | 11457893 |
|  | 1662603 |
|  | 11441071 |
|  | 10878369 |
|  | 14993214 |
|  | 1902591 |
|  | 12102661 |
|  | 11710991 |
|  | 15784555 |
|  | 12396715 |
|  | 15246962 |
|  | 15972639 |
|  | 17015727 |
|  | 17390393 |
|  | 10349636 |
|  | 9348310 |
|  | 16886063 |
|  | 16766576 |
|  | 8990168 |
|  | 15218052 |
|  | 12368207 |
|  | 17570214 |
|  | 9886369 |
|  | 15372109 |
|  | 1545131 |
|  | 12393446 |
|  | 11943142 |
|  | 17441979 |
|  | 15911877 |
|  | 15289500 |
|  | 7544321 |
|  | 9317156 |
|  | 17041003 |
|  | 16428820 |
|  | 10080860 |
|  | 10446807 |
|  | 17097497 |
|  | 15611657 |
|  | 12486099 |
|  | 14688355 |
|  | 9469425 |
|  | 12552499 |
|  | 6305337 |
|  | 11159184 |
|  | 9794653 |
|  | 9725248 |
|  | 12429207 |
|  | 17347451 |
|  | 17173138 |
|  | 12555203 |
|  | 15251440 |
|  | 17210665 |
|  | 11834721 |
|  | 12097365 |
|  | 8529837 |
|  | 1628826 |
|  | 15944226 |
|  | 11700035 |
|  | 16473830 |
|  | 12372981 |
|  | 11427432 |
|  | 9169506 |
|  | 12615659 |
|  | 7650382 |
|  | 16427044 |
|  | 7650381 |
|  | 10580062 |
|  | 7534333 |
|  | 9104810 |
|  | 12088418 |
|  | 12648455 |
|  | 14978096 |
|  | 15908387 |
|  | 17583733 |
|  | 10066343 |
|  | 17209781 |
|  | 12928391 |
|  | 16689860 |
|  | 1698554 |
|  | 11076861 |
|  | 9366559 |
|  | 9916748 |
|  | 15034023 |
|  | 11298489 |
|  | 15947095 |
|  | 14969693 |
|  | 15271900 |
|  | 17318261 |
|  | 15452235 |
|  | 12077239 |
|  | 10799884 |
|  | 16081806 |
|  | 12167712 |
|  | 17117487 |
|  | 16331707 |
|  | 12804771 |
|  | 8378774 |
|  | 8766570 |
|  | 15257986 |
|  | 12097398 |
|  | 2997340 |
|  | 17470541 |
|  | 15944262 |
|  | 15944263 |
|  | 15905520 |
|  | 8228818 |
|  | 12223516 |
|  | 9234781 |
|  | 15905519 |
|  | 16474934 |
|  | 12965263 |
|  | 12874215 |
|  | 16919160 |
|  | 15695523 |
|  | 9094668 |
|  | 7893605 |
|  | 15944275 |
|  | 12219025 |
|  | 12531774 |
|  | 12759426 |
|  | 7590734 |
|  | 6173769 |
|  | 15694411 |
|  | 9916727 |
|  | 1400376 |
|  | 16970687 |
|  | 8696010 |
|  | 7890367 |
|  | 17016559 |
|  | 17557120 |
|  | 17237425 |
|  | 15271961 |
|  | 8228806 |
|  | 9647244 |
|  | 12516540 |
|  | 15271977 |
|  | 12788307 |
|  | 7768593 |
|  | 16959328 |
|  | 16936808 |
|  | 16552032 |
|  | 9916732 |
|  | 7814445 |
|  | 17034586 |
|  | 12389079 |
|  | 16051797 |
|  | 15880044 |
|  | 9551982 |
|  | 15661890 |
|  | 12165521 |
|  | 10362501 |
|  | 12759429 |
|  | 11900986 |
|  | 17656678 |
|  | 15661899 |
|  | 15802553 |
|  | 11991675 |
|  | 14631168 |
|  | 17200147 |
|  | 10202184 |
|  | 16428793 |
|  | 12885563 |
|  | 16552046 |
|  | 15731046 |
|  | 12700631 |
|  | 15728489 |
|  | 10816534 |
|  | 15634895 |
|  | 15009430 |
|  | 12754213 |
|  | 15009431 |
|  | 12070781 |
|  | 9647229 |
|  | 3093809 |
|  | 17237409 |
|  | 10383743 |
|  | 16116187 |
|  | 15799032 |
|  | 17456233 |
|  | 17376993 |
|  | 12093877 |
|  | 14530376 |
|  | 15050961 |
|  | 16889932 |
|  | 8635658 |
|  | 12077263 |
|  | 17026468 |
|  | 7761098 |
|  | 12055229 |
|  | 17015683 |
|  | 11972057 |
|  | 9169744 |
|  | 10220431 |
|  | 10395683 |
|  | 15448160 |
|  | 15331415 |
|  | 17363708 |
|  | 12421979 |
|  | 11854199 |
|  | 11000402 |
|  | 7512988 |
|  | 12914676 |
|  | 17163960 |
|  | 15331418 |
|  | 17148671 |
|  | 9806748 |
|  | 15661037 |
|  | 15210806 |
|  | 16717115 |
|  | 3924594 |
|  | 15514019 |
|  | 15498860 |
|  | 14568937 |
|  | 12588705 |
|  | 10843700 |
|  | 16547278 |
|  | 11592087 |
|  | 12370261 |
|  | 11696572 |
|  | 7750457 |
|  | 17651021 |
|  | 15746081 |
|  | 15660390 |
|  | 9743208 |
|  | 16286661 |
|  | 11854182 |
|  | 11445538 |
|  | 11507186 |
|  | 10207161 |
|  | 12665516 |
|  | 12968017 |
|  | 17142750 |
|  | 11449358 |
|  | 14734640 |
|  | 9199462 |
|  | 7705746 |
|  | 16115485 |
|  | 9300697 |
|  | 7636230 |
|  | 16391178 |
|  | 12370291 |
|  | 17618911 |
|  | 17525184 |
|  | 9144497 |
|  | 17420243 |
|  | 16289099 |
|  | 10499917 |
|  | 12559970 |
|  | 9788810 |
|  | 12447873 |
|  | 9233639 |
|  | 8605935 |
|  | 8387525 |
|  | 14692664 |
|  | 15879135 |
|  | 17634555 |
|  | 16751369 |
|  | 15946645 |
|  | 16210653 |
|  | 14568952 |
|  | 8605933 |
|  | 15320895 |
|  | 15019280 |
|  | 17215524 |
|  | 11786536 |
|  | 16047338 |
|  | 15213264 |
|  | 9862708 |
|  | 9109438 |
|  | 14988089 |
|  | 15630137 |
|  | 12084660 |
|  | 10900344 |
|  | 16987553 |
|  | 16618605 |
|  | 16282454 |
|  | 10561796 |
|  | 9739059 |
|  | 14607904 |
|  | 16717119 |
|  | 17266440 |
|  | 16141073 |
|  | 16141072 |
|  | 11857052 |
|  | 16541469 |
|  | 15719367 |
|  | 11980899 |
|  | 7594565 |
|  | 12579348 |
|  | 6310596 |
|  | 12975380 |
|  | 15544617 |
|  | 16873363 |
|  | 15946996 |
|  | 12890425 |
|  | 16547244 |
|  | 17300840 |
|  | 12507769 |
|  | 14581562 |
|  | 17766435 |
|  | 14738450 |
|  | 16547238 |
|  | 15814744 |
|  | 7818827 |
|  | 16177303 |
|  | 16046307 |
|  | 16368976 |
|  | 14694183 |
|  | 16861661 |
|  | 6427223 |
|  | 2523712 |
|  | 14638787 |
|  | 16861663 |
|  | 8290572 |
|  | 10556835 |
|  | 17049266 |
|  | 11932390 |
|  | 10577740 |
|  | 10477611 |
|  | 16549802 |
|  | 11591796 |
|  | 11970958 |
|  | 9199423 |
|  | 8409521 |
|  | 7635512 |
|  | 14607968 |
|  | 9199420 |
|  | 16723718 |
|  | 11756986 |
|  | 14602571 |
|  | 15845519 |
|  | 11027433 |
|  | 17557376 |
|  | 15774549 |
|  | 2514776 |
|  | 12794718 |
|  | 15383700 |
|  | 12615834 |
|  | 9116217 |
|  | 10899912 |
|  | 16861684 |
|  | 15494513 |
|  | 14970176 |
|  | 7949367 |
|  | 12093672 |
|  | 12441140 |
|  | 11145720 |
|  | 8954615 |
|  | 14568984 |
|  | 15240692 |
|  | 10209035 |
|  | 14970180 |
|  | 10986460 |
|  | 15808517 |
|  | 8809126 |
|  | 16903774 |
|  | 10556424 |
|  | 10666622 |
|  | 12396714 |
|  | 15124210 |
|  | 15299487 |
|  | 15675129 |
|  | 15957503 |
|  | 16672072 |
|  | 3086437 |
|  | 16005068 |
|  | 16081820 |
|  | 16467986 |
|  | 16777275 |

---

|  |  |
| --- | --- |
| Organism | Homo sapiens {Organism urn:agi-taxid:9606} |
|  | Mus musculus {Organism urn:agi-taxid:10090} |
|  | Rattus norvegicus {Organism urn:agi-taxid:10116} |
|  | Homo sapiens |
|  | Mus musculus |
|  | Rattus norvegicus |
|  | Bos taurus |
|  | Danio rerio |
|  | Human |
|  | mouse |
|  | murine |
|  | rat |
|  | void |
|  | pig |
|  | bovine |

---

|  |  |
| --- | --- |
| Mouse chromosome position | 10 66.75 cM |
|  | 10 67.0 cM |

---

|  |  |
| --- | --- |
| OMIM ID | 147570 |
|  | 609135 |
|  | 609532 |
|  | 609423 |
|  | 607948 |
|  | 613254 |
|  | 191100 |
|  | OMIM:147570 |
|  | OMIM:191100 |
|  | OMIM:607948 |

---

|  |  |
| --- | --- |
| Rat chromosome position | 7q22 |

---

|  |  |
| --- | --- |
| Hugo ID | 5438 |
|  | HGNC:5438 |

---

|  |  |
| --- | --- |
| Human chromosome position | 12q15 |
|  | 12q14 |

---

|  |  |
| --- | --- |
| Swiss-Prot Accession | P01579 |
|  | P01579.1 |
|  | P01580 |
|  | P01580.1 |
|  | P01581 |
|  | P01581.1 |
|  | B5BU88 |
|  | Q53ZV4 |
|  | Q542B8 |
|  | Q14609 |
|  | Q14610 |
|  | Q14611 |
|  | Q14612 |
|  | Q14613 |
|  | Q14614 |
|  | Q14615 |
|  | Q8NHY9 |
|  | Q96LA2 |
|  | Q6TDH0 |
|  | IFNG\_MOUSE |
|  | B5BU88\_HUMAN |
|  | IFNG\_HUMAN |
|  | IFNG\_RAT |

---

|  |  |
| --- | --- |
| PIR ID | A01845 |
|  | A93284 |
|  | A01844 |

---

|  |  |
| --- | --- |
| GenBank ID | NC\_000012 |
|  | NM\_000619 |
|  | NP\_000610 |
|  | NG\_015840 |
|  | AC007458 |
|  | AF330164 |
|  | AF375790 |
|  | AAK53058 |
|  | CH471054 |
|  | EAW97180 |
|  | CS723354 |
|  | CAQ43129 |
|  | J00219 |
|  | AAB59534 |
|  | KU759691 |
|  | AB451324 |
|  | BAG70138 |
|  | AB451453 |
|  | BAG70267 |
|  | AF506749 |
|  | AAM28885 |
|  | AM903379 |
|  | CAP17327 |
|  | AY044154 |
|  | AAK95388 |
|  | AY255837 |
|  | AAP20098 |
|  | AY255839 |
|  | AAP20100 |
|  | BC070256 |
|  | AAH70256 |
|  | EF173872 |
|  | ABM53145 |
|  | V00543 |
|  | CAA23804 |
|  | X01992 |
|  | CAA26022 |
|  | X13274 |
|  | CAA31639 |
|  | X62468 |
|  | CAA44325 |
|  | X62469 |
|  | CAA44326 |
|  | X62470 |
|  | CAA44327 |
|  | X62471 |
|  | CAA44328 |
|  | X62472 |
|  | CAA44329 |
|  | X62473 |
|  | CAA44330 |
|  | X62474 |
|  | CAA44331 |
|  | X87308 |
|  | P01579 |
|  | NC\_000076 |
|  | NM\_008337 |
|  | NP\_032363 |
|  | AC153498 |
|  | AY964994 |
|  | CH466578 |
|  | EDL24379 |
|  | AK089574 |
|  | BAC40926 |
|  | AY423847 |
|  | AAR00339 |
|  | BC119063 |
|  | AAI19064 |
|  | BC119065 |
|  | AAI19066 |
|  | EF423643 |
|  | ABN80441 |
|  | FJ617514 |
|  | ACR22509 |
|  | FJ617515 |
|  | ACR22510 |
|  | FJ617516 |
|  | ACR22511 |
|  | K00083 |
|  | AAA37894 |
|  | M28621 |
|  | P01580 |
|  | NC\_005106 |
|  | NM\_138880 |
|  | NP\_620235 |
|  | AC\_000075 |
|  | AABR07057222 |
|  | AAHX01049471 |
|  | AH002184 |
|  | AAA41362 |
|  | CH473960 |
|  | EDM16596 |
|  | X02325 |
|  | CAA26185 |
|  | X02326 |
|  | CAA26186 |
|  | CAA26187 |
|  | X02327 |
|  | CAA26188 |
|  | AF010466 |
|  | AAB66352 |
|  | P01581 |
|  | AMYH02026693 |
|  | AAHY01089300 |
|  | ABBA01026561 |
|  | AC\_000032 |
|  | M29315 |
|  | M29316 |
|  | M29317 |
|  | AC\_000144 |
|  | NC\_018923 |
|  | AABR06049848 |
|  | NT\_029419 |
|  | NW\_001838060 |
|  | NW\_925395 |
|  | AC\_000055 |
|  | Q14609 |
|  | Q14610 |
|  | Q14611 |
|  | Q14612 |
|  | Q14613 |
|  | Q14614 |
|  | Q14615 |
|  | Q8NHY9 |
|  | Q96LA2 |
|  | NT\_039500 |
|  | NW\_001030422 |
|  | Q6TDH0 |
|  | NW\_001084856 |
|  | NW\_047774 |
|  | Q53ZV4 |
|  | 1334287 |
|  | 32694 |
|  | 32692 |
|  | NT\_039501 |
|  | Q542B8 |
|  | 74715697 |
|  | 32690 |
|  | 148692432 |
|  | 15421195 |
|  | 30171281 |
|  | 14030767 |
|  | 184639 |
|  | 126211549 |
|  | 309328 |
|  | 20805896 |
|  | 30171277 |
|  | 26354599 |
|  | 37695615 |
|  | 124479 |
|  | 119617586 |
|  | 32723 |
|  | 124482 |
|  | 124480 |
|  | 74724234 |
|  | 32680 |
|  | 74706461 |
|  | 32688 |
|  | 74706466 |
|  | 74706467 |
|  | 32686 |
|  | 32684 |
|  | 74706462 |
|  | 74706463 |
|  | 32682 |
|  | 74706464 |
|  | 74706465 |
|  | 81911572 |
|  | 149066863 |
|  | 111600468 |
|  | 1124987 |
|  | NW\_000033 |
|  | 204689 |
|  | 111601069 |
|  | 32678 |
|  | 47124535 |
|  | 74740890 |
|  | 2317784 |
|  | NT\_086796 |
|  | 1149600 |
|  | 124476 |
|  | 151415013 |
|  | 163186 |
|  | 6409148 |
|  | 6409149 |
|  | 6409150 |
|  | 75074759 |
|  | AAB64477 |
|  | AAF07862 |
|  | AAF07863 |
|  | AAF07864 |
|  | ABS11696 |
|  | AF140653 |
|  | AF140654 |
|  | AF140655 |
|  | CAA90859 |
|  | EF675629 |
|  | M29867 |
|  | NC\_007303 |
|  | NM\_174086 |
|  | NP\_776511 |
|  | NW\_001495033 |
|  | P07353 |
|  | Q9TQT8 |
|  | Z54144 |
|  | 40363745 |
|  | 47678933 |
|  | 55962522 |
|  | 82078276 |
|  | 82092931 |
|  | AB126869 |
|  | AB158361 |
|  | BAD06253 |
|  | BAD20663 |
|  | BX465210 |
|  | CAI11947 |
|  | DU644822 |
|  | NC\_007115 |
|  | NM\_212864 |
|  | NP\_998029 |
|  | NW\_001513233 |
|  | Q5RHV5 |
|  | Q75S22 |
|  | M29383 |
|  | AAA36124 |
|  | XM\_125899 |
|  | XP\_125899 |
|  | NW\_044022 |
|  | NT\_029419.8 |
|  | 47086308 |
|  | 126007509 |
|  | 47086309 |
|  | 405790 |
|  | Dr.90633 |
|  | ZDB-GENE-040629-1 |
|  | dre:405790 |

---

|  |  |
| --- | --- |
| Swiss-Prot ID | IFNG\_RAT |
|  | IFNG\_HUMAN |
|  | IFNG\_MOUSE |
|  | B5BU88\_HUMAN |

---

|  |  |
| --- | --- |
| Cell Localization | Secreted |
|  | Extracellular |

---

|  |  |
| --- | --- |
| Ensembl ID | ENSG00000111537 |
|  | ENSP00000229135.3 |
|  | ENST00000229135.4 |
|  | ENSMUSG00000055170 |
|  | ENSMUSP00000063800.3 |
|  | ENSMUST00000068592.4 |
|  | ENSRNOG00000007468 |
|  | ENSRNOP00000009917.1 |
|  | ENSRNOT00000009919.2 |
|  | ENST00000229135.3 |
|  | ENSP00000229135 |
|  | ENST00000229135 |
|  | ENSMUSP00000063800 |
|  | ENSRNOT00000009919 |
|  | ENSMUST00000068592 |
|  | ENSRNOP00000009917 |

---

|  |  |
| --- | --- |
| MGI ID | MGI:107656 |
|  | 107656 |

---

|  |  |
| --- | --- |
| RGD ID | 2866 |

---

|  |  |
| --- | --- |
| Unigene ID | Hs.856 |
|  | Mm.240327 |
|  | Rn.10795 |
|  | Bt.188 |
|  | Dr.90633 |
|  | Mm.529 |

---

|  |  |
| --- | --- |
| Homologene ID | 55526 |

---

|  |  |
| --- | --- |
| IPI ID | IPI00307209 |
|  | IPI00214504 |
|  | IPI00136892 |

---

|  |  |
| --- | --- |
| Microarray ID | 99334\_at |
|  | 1370790\_at |
|  | M29317\_at |
|  | AF010466\_s\_at |
|  | A\_43\_P13320 |
|  | NM\_138880 |
|  | 1425947\_at |
|  | k00083\_s\_at |
|  | Msa.800.0\_at |
|  | A\_51\_P220976 |
|  | M28621 |
|  | A\_65\_P14475 |
|  | K00083.1 |
|  | X13274 >2 |
|  | X13274 >1 |
|  | 40702\_at |
|  | 1021\_at |
|  | 1611\_s\_at |
|  | 210354\_at |
|  | J00219\_s\_at |
|  | v00536\_rna1\_at |
|  | A\_23\_P151294 |
|  | I\_932362 |
|  | A\_52\_P68893 |
|  | A\_14\_P126642 |

---

|  |  |
| --- | --- |
| Homo sapiens Chromosome position | 12q14 |

---

|  |  |
| --- | --- |
| Ensembl Transcript ID | ENST00000229135 |

---

|  |  |
| --- | --- |
| FunctionalClass | cytokine |
|  | interferon-gamma receptor binding |
|  | transcriptional activator |
|  | transcription activator |

---

|  |  |
| --- | --- |
| Bos taurus Chromosome position | 5q22-q24 |

---

|  |  |
| --- | --- |
| Mus musculus Chromosome position | 10 67.0 cM |

---

|  |  |
| --- | --- |
| KEGG pathway | Type I diabetes mellitus |
|  | Cytokine-cytokine receptor interaction |
|  | Jak-STAT signaling pathway |
|  | Natural killer cell mediated cytotoxicity |
|  | TGF-beta signaling pathway |
|  | Regulation of autophagy |
|  | T cell receptor signaling pathway |

---

|  |  |
| --- | --- |
| RefSeq ACCNUM | NP\_000610 |

---

|  |  |
| --- | --- |
| Rattus norvegicus Chromosome position | 7q22 |

---

|  |  |
| --- | --- |
| Ensembl Gene ID | ENSG00000111537 |

---

|  |  |
| --- | --- |
| Keyword | desmoplakin |
|  | matrix |
|  | antigen |
|  | interferon |
|  | tumor necrosis factor |
|  | nitric oxide synthase |
|  | chemokine |
|  | cytokine activity |
|  | host |
|  | endotoxin |

---

|  |  |
| --- | --- |
| LocatorString | digtext:C:\Users\Ariadne\Customers\Elan\Elan weekly report from literature condensed.txt |

---

|  |  |
| --- | --- |
| Proteome Organismal Role | Cell migration/motility |
|  | Anti-pathogen response |
|  | Cell-to-cell signalling |

---
